# Social Dysfunction in Psychosis Is More Than a Matter of Misperception: Advances From the Study of Metacognition

**DOI:** 10.3389/fpsyg.2021.723952

**Published:** 2021-10-14

**Authors:** Paul H. Lysaker, Ilanit Hasson-Ohayon, Courtney Wiesepape, Kelsey Huling, Aubrie Musselman, John T. Lysaker

**Affiliations:** ^1^Department of Psychiatry, Richard L. Roudebush VA Medical Center, Indianapolis, IN, United States; ^2^Department of Psychiatry, Indiana University School of Medicine, Indianapolis, IN, United States; ^3^Department of Psychology, Bar-Ilan University, Ramat Gan, Israel; ^4^Department of Psychology, Indiana State University, Terre Haute, IN, United States; ^5^School of Psychological Sciences, University of Indianapolis, Indianapolis, IN, United States; ^6^Department of Philosophy, Emory University, Atlanta, GA, United States

**Keywords:** psychosis, Schizophrenia, self, intersubjectivity, psychosocial function, metacognition, social cognition, theory of mind

## Abstract

Many with psychosis experience substantial difficulties forming and maintaining social bonds leading to persistent social alienation and a lack of a sense of membership in a larger community. While it is clear that social impairments in psychosis cannot be fully explained by symptoms or other traditional features of psychosis, the antecedents of disturbances in social function remain poorly understood. One recent model has proposed that deficits in social cognition may be a root cause of social dysfunction. In this model social relationships become untenable among persons diagnosed with psychosis when deficits in social cognition result in inaccurate ideas of what others feel, think or desire. While there is evidence to support the influence of social cognition upon social function, there are substantial limitations to this point of view. Many with psychosis have social impairments but not significant deficits in social cognition. First person and clinical accounts of the phenomenology of psychosis also do not suggest that persons with psychosis commonly experience making mistakes when trying to understand others. They report instead that intersubjectivity, or the formation of an intimate shared understanding of thoughts and emotions with others, has become extraordinarily difficult. In this paper we explore how research in metacognition in psychosis can transcend these limitations and address some of the ways in which intersubjectivity and more broadly social function is compromised in psychosis. Specifically, research will be reviewed on the relationship between social cognitive abilities and social function in psychosis, including measurement strategies and limits to its explanatory power, in particular with regard to challenges to intersubjectivity. Next, we present research on the integrated model of metacognition in psychosis and its relation to social function. We then discuss how this model might go beyond social cognitive models of social dysfunction in psychosis by describing how compromises in intersubjectivity occur as metacognitive deficits leave persons without an integrated sense of others' purposes, relative positions in the world, possibilities and personal complexities. We suggest that while social cognitive deficits may leave persons with inaccurate ideas about others, metacognitive deficits leave persons ill equipped to make broader sense of the situations in which people interact and this is what leaves them without a holistic sense of the other and what makes it difficult to know others, share experiences, and sustain relationships. The potential of developing clinical interventions focused on metacognition for promoting social recovery will finally be explored.

## Introduction

Substantial difficulties forming and maintaining social bonds have been repeatedly observed among those diagnosed with psychosis (Macdonald et al., 2000; Bratlien et al., 2013). Relationships with romantic partners, family, friends, and others may be confusing, conflict laden, and unstable, in both the moment and for the long term (Kurs et al., 2005; Palumbo et al., 2015). Consistent with this, many diagnosed with psychosis report that social alienation and an attenuated sense belonging or membership in a larger community are chief concerns (Ritsner and Grinshpoon, 2015; Rowe and Davidson, 2016; Leonhardt et al., 2017). Importantly, decrements in social function are also linked with a range of other negative outcomes, including mortality (Pantell et al., 2013; Degnan et al., 2018).

Nevertheless, the phenomena which cause or sustain disturbances in social function in psychosis are not clearly understood. It has been established that symptoms or other traditional features of psychosis cannot fully explain social impairment (Strauss and Carpenter, 1974). Across studies, social function appears to become less stable prior to the onset of diagnosable signs of psychosis (McGorry et al., 1995; MacDonald et al., 2005; Masse et al., 2020) and may cause or exacerbate symptoms rather than the reverse (Kidd, 2012). One recent proposal is that deficits in social cognition are a root cause of social dysfunction (Couture et al., 2006). For example, it has been suggested that social relationships become untenable when persons diagnosed with psychosis misperceive what others feel, think or desire as a result of deficits in social cognitive domains such as Theory of Mind and affect recognition (Pinkham, 2014). Specifically, with failures to accurately grasp different aspects of others' experiences during social encounters persons may lack the kind of information needed to guide adaptive psychological and behavioral reactions to social circumstances.

While there is evidence supporting this view, there are also limitations to this view. Research does suggest that many with psychosis struggle to correctly identify the emotional and cognitive states of persons in stories, photographs and video clips, and that poorer performance on these tasks is correlated with greater deficits in psychosocial function (Pinkham et al., 2018; Green et al., 2019). However, many with psychosis have social impairments without accompanying deficits in social cognition (James et al., 2018). They also report feeling disconnected from others (Leonhardt et al., 2017; Hasson-Ohayon et al., 2020) in a way that implies challenges to intersubjectivity that go beyond the misperception of what others think, feel and want.

One avenue of research that has sought to transcend these limitations and offer a potentially more nuanced understanding of the phenomena which shape social dysfunction in psychosis has focused on the relationship of the metacognitive deficits with interpersonal function (Lysaker et al., 2020b). In this work, metacognition refers to the processes that enable persons to notice and make sense of their own and other's embodied, cognitive and emotional states, both within the moment and over the course of a life (Lysaker and Lysaker, 2020). In contrast to social cognition, metacognition is concerned with the degree to which information is integrated, rather than the accuracy or speed with which a particular conclusion is reached (Lysaker and Hasson-Ohayon, 2014). Pertaining to social function in general, intact metacognition facilitates the integration of information into a contextualized and holistic sense of self and others which allows for the evolution and sustenance of the kinds of mutual understanding with others often reported as lacking in psychosis (Lysaker et al., 2020a). Beyond allowing for an awareness of a particular aspect of others' experiences, intact metacognition enables persons to form a sense of their and others' places within the larger community, allowing for a sense of belonging in larger social groups, another thing often missing in psychosis.

To explore the possibility that deficits in metacognitive capacity may offer unique insights into the development and sustenance of compromises in interpersonal function in psychosis, this paper will first review research on the relationship between social cognitive abilities and social function in psychosis. Definitions, measurement strategies, supporting research, and the limitations of a model of deficits in social cognition as an explanation for social dysfunction in psychosis will be explored. Next, we will present research on the integrated model of metacognition in psychosis and its relation to social function. We then discuss how this model may go beyond social cognitive models of social dysfunction in psychosis by describing how compromises in intersubjectivity occur as metacognitive deficits leave persons without an integrated sense of others' purposes, places in the world, possibilities and personal complexities. Implications for the development of clinical interventions that promote the recovery of social connections will finally be explored.

## Social Cognition and Disturbances in Social Functioning

### Research Methods and Findings

Social cognition refers to a group of mental operations that allow persons to attend to and judge various aspects of social interactions (Green et al., 2008). It is theorized to have four core domains: Theory of Mind (ToM), social perception, attribution bias, and emotional processing (Pinkham et al., 2014). Each of these are commonly assessed with tasks that require the demonstration of that skill rather than a self-report of a one's own ability to competently make judgements in these areas (Couture et al., 2006). To date the largest effort to codify these approaches has been the Social Cognition Psychometric Evaluation (SCOPE; Pinkham et al., 2014). These studies have identified three measures that demonstrate the strongest psychometric properties including a measure of ToM, The Hinting Task (Corcoran et al., 1995), and two measures of emotion processing, the Bell Lysaker Emotion Recognition Task (BLERT; Bell et al., 1997), and the Penn Emotion Recognition Task (ER-40; Kohler et al., 2003) (Pinkham et al., 2018). The Hinting Task measures a person's capacity to infer intentions from indirect speech or verbal “hints” (e.g., Lindgren et al., 2018; García-Fernández et al., 2020). For example, what does a person in a fictional scenario want to convey when they say a certain thing. The BLERT measures the ability to correctly identity which of seven emotional states an actor is portraying in a short video clip (Bell et al., 1997). Similarly, the ER-40 is a computer-based task that measures the ability to accurately identify which of five possible facial emotions are being expressed in a still photograph (Kohler et al., 2003).

Other prominent measures of social cognition include the Faux Pas Recognition Test (Stone et al., 1998), a story-based task in which individuals must correctly identity a social faux pas and the Reading the Mind in the Eyes Test (Baron-Cohen et al., 2001), a measure in which individuals must correctly identify the correct emotional state expressed from a photograph of one set of eyes. Another measure is the Movie for the Assessment of Social Cognition (Dziobek et al., 2006). In this task individuals watch a video of a dinner party and answer multiple choice questions about the thoughts, feelings, and intentions of characters, which includes opportunities for them to detect irony and sarcasm in the speech of the characters. Each of these measures assess whether individuals can correctly identify the intentions, emotions, or thoughts of others using controlled visual stimuli, like photos or videos.

As operationalized within these tasks, deficits in social cognition have been proposed as a primary cause of social dysfunction in psychosis (Green et al., 2019). For example, frequently mistaking someone with benevolent intentions as planning to be aggressive would presumably undermine newly emerging or long-standing relationships. Similarly, mistaking another's surprise as anger could lead to misunderstanding, mutual distrust and confusion. Even less dramatic errors when guessing what someone wants on the basis of how he or she is behaving might derail the ability to meet that person's needs and cooperate with him or her.

Evidence that deficits in social cognition underlie social dysfunction comes from multiple sources. Samples of persons diagnosed with clinical high risk (Davidson et al., 2018), early psychosis, and chronic psychosis (Valaparla et al., 2017) perform significantly worse in multiple domains of social cognition relative to persons without psychosis (Savla et al., 2013). Other studies have found that persons with psychosis experience difficulties understanding implied social cues, comprehending implicit information, explaining metaphors, and interpreting humor (Pawełczyk et al., 2018). Importantly, poorer performance on measures of social cognition has been linked to deficits in social functioning, social skills, and community integration (Couture et al., 2006; Fett et al., 2011). More specifically, deficits in affective prosody recognition have been found to be significantly associated with social functioning (Bonfils et al., 2019b), while Gardner et al. (2019) have reported an association between better emotion recognition and social functioning. Finally, persons with intact social cognition have reported higher levels of social satisfaction in their life (Mike et al., 2019).

### Limitations

There are limitations to explaining social dysfunction in psychosis as the result of deficits in social cognition. While deficits in social cognition have been observed among persons diagnosed with psychosis, they are not ubiquitous. Significant proportions of persons diagnosed with psychosis do not have significant deficits in social cognition (James et al., 2018). Other studies not only have failed to find a positive link between social cognition and social function but have found that poorer social perception and emotion processing abilities are associated with better engagement in recreational and prosocial activities (Woolverton et al., 2018). Other studies have suggested that deficits in emotion recognition might be a way to protect persons from the pain of social dysfunction rather than a singular cause of that dysfunction (Hasson-Ohayon et al., 2017, 2019).

Beyond this, research has not consistently found that improvements in social cognition affect social function. A non-randomized study of Social Cognitive Skills Training (SCST), a group-based social cognition intervention, led to both improved social cognition and social functioning (Lim et al., 2020). However, consistent with other reviews (Javed and Charles, 2018; Tan et al., 2018), a randomized trial of Social Cognition and Interaction Training (SCIT) did not find greater changes in social skills compared to a control group (Gordon et al., 2018). Similarly, cognitive remediation approaches that include social cognitive training have demonstrated improvements in social cognition but not social functioning (Vidarsdottir et al., 2019). Recognizing the need to enhance the generalizability of these interventions to functional outcomes, Horan et al. (2018) introduced six *in vivo* training sessions in community settings to Social Cognitive Skills Training (SCST). They again found no significant changes in functional capacity or real-world functioning.

Considered in a larger theoretical frame, social cognitive deficits are not a fully satisfying explanation for social dysfunction, since accurate accounts of others' thoughts, feelings, and intentions does not necessarily lead to healthy social functioning in general. Focusing on one domain of social cognition, Plastow (2012) has suggested that Theory of Mind is “a poor and distant account” of persons' relationships with one another” (p. 292). In his view, Theory of Mind fails to capture how we know each other in contexts that involve our having pre-existing feelings or ideas about the other. It also does not explain the frequently stormy nature of human relationships, since many with presumably intact social cognitive abilities experience difficulties relating to others. Cole and Millett (2019) further suggest that there is evidence that people do not routinely form the kinds of specific formal representations that are assessed by tests of social cognition such as identifying singular emotions or motives. In a parallel critique of social cognitive approaches to autism, Gallagher (2004) notes: “the external challenge to the theory of mind account of autism, then, can be stated clearly: deficits in theory of mind cannot explain autism because the theory of mind itself is not a good explanation of non-autistic intersubjective experience” (p. 201).

Beyond the empirical and conceptual issues noted above, a larger problem for any social cognitive model of social dysfunction is its lack of concordance with first person and clinical accounts of the phenomenology of psychosis. Qualitative studies do not generally affirm that persons diagnosed with psychosis experience themselves as misunderstanding others. Persons diagnosed with psychosis instead describe the common experience of being emotionally distant from, unconnected to and misunderstood by other people (Davies et al., 2019; Nilsson et al., 2019) and their larger community (Saks, 2008; Bromley et al., 2013; Townley, 2015). They find sharing experience and establishing intimacy with others to be extraordinarily challenging in ways that go well beyond a sense of misunderstanding others' thoughts and emotions.

These reports are consistent with older psychoanalytic observations that persons diagnosed with psychosis experience social interactions as frightening and overwhelming (Fromm-Reichmann, 1954; Searles, 1965; Pec et al., 2020; Ridenour et al., 2020). From an existential perspective, Laing (1978) suggested the experience of psychosis involved a limited sense of self whose minimal coherence was threatened by intimacy. Rogers (1967) also reported that therapists' overtures of warmth and empathy were often intolerable and overwhelming to his patients. More recently, phenomenological models have suggested that in psychosis there are limitations in the ability to form a shared sense of embodied and social experience with others (Fuchs, 2015; Henriksen and Siersbæk Nilsson, 2017; Phulpin et al., in press). These challenges also have been recently reported in broad surveys of clinicians (Moskalewicz et al., 2021).

### Social Cognition, Intersubjectivity, and Social Function in Psychosis

One way to characterize the aspects of social dysfunction which do not match a social cognitive model are as disturbances in intersubjectivity. Intersubjectivity refers to a continuum of interactions among at least two persons (Beebe et al., 2005; Cortina and Liotti, 2010) that allow for shared understanding of emotional, cognitive and embodied experiences to develop (Benjamin, 1990; Trevarthen, 1998; Stern, 2000). Intersubjectivity is thought to begin in early infancy (Trevarthen, 1998) as a result of a reliable and safe bond with a caretaker (Fonagy, 1991), as well as intact underlying neural systems that simulate others' experiences (Gallese, 2003). It is believed to first involve the emergence of a preverbal subjective sense of self and the caregiver (Stern, 2000) and then to develop alongside language, becoming the basis for reflection upon the experience of oneself and others (Stets and Burke, 2000; Cortina and Liotti, 2010).

Following the work of Merleau-Ponty (1948/1992), we use the term intersubjectivity to describe what enables persons to form a sense of another person as a whole which cannot be grasped by consideration of any of its elements on their own. Intersubjectivity is both an active and passive process that involves the immediate awareness of the presence of another person and one's relationship to that person (Ollagnier-Beldame and Coupé, 2019). Intersubjectivity allows persons to be understood as more than “a multitude of details that one subsequently puts together to create meaning, but rather as a meaningful whole first—an intentional consciousness with an experience of its own unique subjectivity” (Pienkos, 2015, p 195). Intersubjectivity, as is inherent in the term itself, is something that happens between persons or subjectivities and not within one or the other alone. Intersubjectivity emerges through a joint understanding that occurs between persons through actual or potential dialogue (e.g., Buber, 1970; Dewey, 1981). It involves the mutual recognition by two persons of each other that allows for the awareness of differences between those persons (Benjamin, 1990) and represents a shared understanding of another person who also is trying to understand the person who is trying to understand them. These shared understandings are finally something that may both linger and evolve. The sense a person forms about another intersubjectively may serve as a context for understanding what transpires in a future interaction while also being subject to revision as the relationship with that other person develops.

Returning to the issue of the factors that contribute to social dysfunction in psychosis, an understanding of challenges to intersubjectivity in psychosis makes plain the limits of a social cognitive model. An incorrect *theory* of another person's thoughts and feelings, that might arise from social cognitive deficits could in part thwart the development of a shared understanding. For example, mistakenly believing one person's tears are sadness rather than anger, or that someone's facial expression suggests they want help rather than be left alone, might complicate the development of a shared, holistic sense of that person. However, knowing whether one is seeing tears of sadness, joy, or rage is not the same as what is entailed in having a holistic sense of that person. In fact, one could correctly identify a person's emotions or intentions but understand very little about who that person is as a unique being. In parallel, one could be mistaken about a particular aspect of another's emotional experience, but still form a larger picture that adequately captures broader aspects of that person, such that someone might still have a larger working sense of another person even though some of the details (e.g., the meaning of tears or a facial expression) do not match the other's experience of them. Thus, a social cognition model of social dysfunction as operationalized above cannot fully account for difficulties establishing intersubjectivity in psychosis.

## Metacognition and Disturbances in Social Functioning

### Intersubjectivity, Metacognition and Social Function in Psychosis

While social cognitive deficits may not go far enough in explaining how efforts to relate to others intersubjectively could be challenged in psychosis, compromises in another form of cognition, metacognition, might extend our understanding of these challenges. Across psychological disciplines, the concept of metacognition has been used to refer to persons' awareness of their thoughts and feelings and how they adjust their thoughts and actions as a result (Flavell, 1979; Semerari et al., 2003; Moritz and Lysaker, 2018). In research on disturbances in subjectivity in psychosis, metacognition has been used to operationalize the processes that enable a reflexive awareness of oneself and others (Lysaker and Lysaker, 2020; Lysaker et al., 2020b). Specifically, this work has used an integrative model of metacognition that considers metacognition as the spectrum of activities that allow persons to become aware of specific cognitive, emotional and bodily experiences and to integrate these into a broader and evolving sense of their and others' unique identities and places in the world (Lysaker and Hasson-Ohayon, 2021). As reflective activities proceed, metacognitive acts are understood as intimately coupled with, informed by and informing, actions taken by the person in the world in response to emerging psychosocial challenges and possibilities (Lysaker and Dimaggio, 2014; Lysaker et al., 2020a). In this model, intact metacognitive capacities are thought to make available a contextualized and integrated sense of self and others whereas deficits in metacognitive capacity contribute to experiences of self and others that are increasingly fragmented.

Key for the study of social function in psychosis are at least two features of this model. The first is that metacognition is always an intersubjective activity (Hasson-Ohayon et al., 2020), such that any sense formed about oneself or others is always done so with an actual or potential partner who can share that sense (Lysaker and Lysaker, 2020). Second, in this model sense of self and sense of others, while naturally related, can be measured separately. Concretely, this is reflected in the structure of the Metacognition Assessment Scale—Abbreviated (MAS-A; Lysaker and Klion, 2017).

The MAS-A consists of four scales: Self-reflectivity (S), Understanding the mind of the other (O), Decentration (D) and Mastery (M) and is rated on the basis of a narrative interview or extended speech sample. Each scale contains multiple items, each of which is an individual metacognitive act. Items of the S, O and D scales are in order of the degree to which integration is required for their successful engagement, with each item requiring a more complex act than the previous item. For example, the fifth item of the S scale, which involves persons noticing that their emotions and subjective experiences are shifting, requires that persons can perform the act right before which is the ability to notice different nuanced emotions. Thus, a person judged insufficiently able to perform the metacognitive act described by a given item would be judged unable to perform any more complex acts on that scale. For M scale, which measures the use of metacognitive knowledge to frame and respond to challenges, items require increasingly complex levels of integration. Higher scores on any given scale thus reflect more integrated as opposed to more fragmented experience.

Of the four MAS-A subscales, two are directly concerned with forming thoughts about others and so may be especially relevant for understanding social dysfunction in psychosis. The first, O, is an eight-point scale (0–7) with higher scores reflecting more integrated senses of specific others while lower scores reflect more fragmented senses of other, specific people. For example, a score of “3” on this scale would suggest awareness of the different thoughts of another person but little to no sense of the emotions they feel, intentions they are trying to communicate or any concrete sense of that person across time. By contrast, a score of “6” should reflect awareness of others having thoughts and feelings that are influencing them in a particular way in a unique moment. The second scale, D, is a four-point scale (0–3). It assesses persons' sense of their relationship to a community of other people with their own distinct point of view and values. Lower scores indicate a less integrated sense of one's distinct place in the community and higher scores indicate a more integrated sense of what ties and distinguishes one from others. As an example, a score of “0” on this scale would reflect an absence of an awareness of events in the larger world not directly related to the individual and little to no sense that others can and do see the world in legitimately different ways from oneself. By contrast, a score of “3” would suggest awareness of how others see the world from unique perspectives and that others are living their lives and relating to others in ways that are unique to them.

The MAS-A O and D scales thus assesses awareness of other people in several ways that deviate from the social cognition measures described above. Because higher scores on O and D reflect increasingly complex, integrated ideas but not necessarily a specific, accurate judgement, a person might make an erroneous guess about another person's emotional state or intentions but have a complex, holistic sense of that person and so receive a higher score on the MAS-A. Similarly, another person might make correct guesses about the emotions and intentions of another but have little to no larger sense of that person as a unique being with a unique history and therefore be assigned a lower score on the MAS-A. Procedurally, these rating also differ from most methods for measuring social cognition. Since, MAS-A ratings are derived from how information is assembled as persons talk about potentially momentous events in their lives rather than how they perform impersonal laboratory tasks, the MAS-A may also uniquely capture something that occurs when persons think about things which are personally salient and interpersonally meaningful.

The MAS-A has demonstrated adequate psychometric properties (Lysaker et al., 2014) and has been used in a range of international studies to confirm that persons in different phases of psychosis experience metacognitive deficits (Lysaker et al., 2020b). These studies have also confirmed that metacognition, as expected, interacts with different forms of cognition, including social cognition and neurocognition in the unique manner of a central node in a larger network (Hasson-Ohayon et al., 2018). As in the case of social cognition, the potential link with social function and metacognition in psychosis has been supported in a range of ways. Poorer metacognitive capacities have been related to the self-report of making fewer attempts to reach out to others for support (Kukla et al., 2013) as well as to the abilities which underpin the perception of events from alterative perspectives (Lysaker et al., 2007b). Among persons with first episode psychosis, greater levels of metacognitive deficits have been linked with a history of a less stable social network (Masse et al., 2020), perceived social support (Masse and Lecomte, 2015) and functional outcome (Davies et al., 2017).

In terms of specific metacognitive capacities, deficits in the MAS-A O subscale have been uniquely linked to lesser levels of self-compassion (Hochheiser et al., 2020), reduced empathy (Bonfils et al., 2019a) and emotional withdrawal from others (Lysaker et al., 2005). Lower scores on D have similarly been linked to a weaker sense of intimate connection with others (Fischer et al., 2020). In this same study, higher levels of overall metacognitive function were found to moderate the effects of symptom severity on the experience of intersubjectivity, such that with better metacognitive functioning, symptoms were not found to affect social function as significantly. Lower scores on the MAS-A M scale have also been linked to reduced abilities to respond adaptively to challenges in relationships with unique deficits in Mastery being linked to heighted social anxiety (Lysaker et al., 2011b) and poorer therapeutic alliance (Davis et al., 2011). In a longitudinal study, poorer Mastery has also been linked to fewer social contacts and social resources over time after controlling for initial levels of social function (Lysaker et al., 2011a).

Supporting the possibility that metacognition may add something to our understanding of social dysfunction, beyond what is explained by social cognition, factor scores reflecting metacognitive function have been reported to be more closely related to the frequency of social contacts and basic capacities for interpersonal relatedness than a parallel social cognition factor (Lysaker et al., 2013). Other work has found metacognitive capacity to be more closely related to social quality of life than social cognition (Hasson-Ohayon et al., 2015).

### Understanding Social Dysfunction in Psychosis Through the Lens of Metacognition

As noted above, deficits in social cognition have been one of the most prominently proposed sources of impairments in social function in psychosis. There is, however, a limit to their explanatory power when considering challenges to intersubjectivity that leave persons bereft of lasting intimate connections and a sense of community membership. Metacognitive deficits, as reviewed above, may fill this gap and advance our understanding of social dysfunction in several ways.

First, metacognitive research makes visible an architecture composed of dynamic, interacting mental activities which supports intersubjectivity, activities which if compromised could undermine the potential for intersubjectivity. Concretely the metacognitive processes operationalized in the MAS-A O and D subscales describe how persons, to varying degrees, form holistic, contextualized ideas about others as unique beings with whom their personal experience could be shared. As persons form ideas about themselves, others and the world, metacognitive processes simultaneously allow for a sense of the persons who are and could be reacting to those ideas. Metacognitive processes enable a lattice of experiences which includes an ongoing and evolving sense of the other person one is relating to, and of one's and the other's reactions to those exchanges, making ongoing shared understanding possible. When metacognition then is compromised, as seen in psychosis, a person would be left with a reduced sense of the other in a social exchange, limiting any potential for a sense of connection or joint membership in a larger community.

As an illustration, to form an intimate bond with the person in our previous example who had been crying or making a certain facial expression, one would have to have a holistic sense of that person who was crying or making those expressions. With metacognitive compromise one might see or not see the meaning behind the tears or facial expression, but regardless see the other as “a multitude of details,” and there could little more than the potential for superficial cooperation. Concretely, the others' subtle reaction in this example would be relatively indiscernible, regardless of social cognitive abilities, leading to a vicious cycle in which intersubjectivity could be experienced as threatening and the experience of self and others would remain fragmented.

To express this point metaphorically, social cognition allows us to grasp the bricks in a social interaction but not the mortar. Social cognition allows us to know a particular thing about another but not how those things relate to one another in a complex, contextualized, and ongoing manner. Metacognition, on the other hand describes how those bricks are related to one another and to the broader world. As a construct, it also allows us to understand why, if the relations among those bricks are disrupted, an unstable structure is left.

In terms of what metacognition reveals about that mortar, or about the different elements of experience whose combinations make up our sense of others and ourselves as unique beings, we suggest the construct of metacognition makes at least four things visible. As described in the beginnings of existentialism (Nietzsche, 1886/1966; Kierkeegard, 1983/1849), and psychology (James et al., 1890) as well as in constructivist psychology (Kelly, 1964), people experience themselves living and acting in the world in pursuit of certain things and in the company of others who have their own pursuits and perspectives. Addressing challenges to intersubjectivity through the lens of metacognition does not fall back into a kind of dualism that believes challenges to relationships with others takes place within single, solitary minds. Instead, social connections are possible and challenges to them take place across the flow of lives within a world that includes other persons oriented toward similar and different purposes and possibilities, and situated or positioned within larger communities and at particular times in history (Lysaker and Lysaker, 2020). Part of the mortar of intersubjectivity thus concerns an evolving and contextualized awareness of these purposes, possibilities and positions as they pertain to others in the world.

Intact metacognition allows for a fourth kind of sense of the other, namely, as always having or expressing different qualities or parts of themselves in any given context. These qualities may be complimentary, contradictory or unrelated, and most will come and go depending on the situation and evolve over time. Metacognition allows us to maintain a coherent, evolving sense of the other as they, for example, are more aggressive, vulnerable or self-centered on different days. With metacognitive function, we thus have a capacity that, if degraded, theoretically could erode a persons' sense of another's purposes, possibilities, positions, as well as of the diverse and dynamic nature of the other's character as a unique person, creating the likelihood of a lack of mutual understanding and alienation in a manner that matches first person reports as well as phenomenological observations in psychosis (Saks, 2008; Townley, 2015; Leonhardt et al., 2017; Nilsson et al., 2019). Altogether then, decrements in metacognition threaten social bonds because they make it difficult to relate to others in terms of their contextualized purposes and to form shared understandings about possibilities that emerge within social interactions.

In a related point, metacognitive research also allows us to see how awareness of the other, as well as the successful establishment of intersubjectivity, could theoretically require multiple domains of metacognitive function rather than just a single intact monolithic function. Specifically, we need self-reflectivity working alongside awareness of the other as well as decentration and mastery to jointly generate a sense of another person's experience. For example, to form a sense of another person's experience of a particular loss or remarkable achievement one may need to be able to form a sense of one's own experience of similar circumstances or to recognize how one has never experienced anything similar (Dimaggio et al., 2008). Self-reflectivity is also needed to make sense of changes in other people while adjusting as errors are detected. This may be easily seen when encountering another person who is acting out of character. One has to form a sense of whether the unexpected actions represent changes in the person or if the person “is not himself or herself today.” This again may require an appreciation of times in the past when one has acted anomalously. With healthy metacognitive function one can acknowledge this, recalling one's own past mistakes, and recognizing one's general fallibility. Mastery may also be needed here as it allows the cultivation of humility when facing one's own limitations when trying to understand others. And if we return to compromised capacities, we can also see that if there are limited levels of metacognitive mastery, there might be little to do in the face of distress and hence little reason to relate to others or form cooperative bonds with them. That is, with reduced mastery there may be little to no sense of personal agency and less reason to try to make sense of what others want, think and feel.

As noted above, intact social cognitive abilities do not guarantee a harmonious social life. In a similar manner, intact metacognitive function is not necessarily a road to healthy social connections. However, intact metacognition would seem necessary but not sufficient for a sense of community membership. In particular, decentration is necessary to be able to perceive a world in which persons have differing roles, pursuits and are connected in ways that go beyond individual needs and consider the communal good. Similarly, at higher levels, the construct of mastery involves the consideration of the unique needs of persons and the larger community when deciding how to live with particular challenges and pursue future possibilities. Thus, metacognitive abilities, because they involve more than perceiving discrete facets of discrete persons, allow for broader understandings which support prosocial behavior.

### Addressing Social Dysfunction in Psychosis Through the Lens of Metacognition

Metacognitive research in psychosis also has direct implications for treatment. If metacognitive compromise results in profound disturbances in a person's sense of connection with others, then treatments which address metacognitive deficits should lead to opportunities for the growth of social relationships and community membership. Concretely, if treatment can enhance self-reflectivity, awareness of others, mastery and decentration, then it would be expected that unique purposes, possibilities and positions experienced by persons would become clearer, thereby enriching intersubjective experience.

One emerging method for addressing metacognition in psychosis is an integrative form of individual psychotherapy referred to as Metacognitive Reflection and Insight Therapy (MERIT; Lysaker and Klion, 2017). Developed iteratively alongside the metacognitive research presented above, MERIT explicitly seeks to enhance the metacognitive domains of Self-reflectivity, Awareness of the other and Mastery, leading to enhancement in Decentration. In a manner roughly analogous to physical therapy, metacognitive capacity is thought to improve as patients engage in metacognitive acts which meet but do not exceed their current, maximal metacognitive capacity. Though still in its infancy, randomized trials, open trials, qualitative and case studies on this approach have suggested that this treatment is acceptable to patients, leads to clinically meaningful gains and have linked this approach to growth in metacognitive capacity, agency, historicity, compassion and greater potential for future action (Lysaker et al., 2020c). Particularly relevant to the issue of intersubjectivity, a recent MERIT trial has found that clinical gains are most closely related to interventions which promote reflection on interpersonal processes, including that of the patient being known by the therapist (Lavi-Rotenberg et al., 2021). In parallel, a metacognitive oriented social skills training program was found to be effective in a randomized trial that resulted in concurrent improvements in metacognitive capacity and interpersonal function (Inchausti et al., 2018).

Importantly, however, metacognitive research by no means suggests that only one approach to treatment could promote metacognition or social recovery. To the contrary, this work suggests a range of basic conditions for the promotion of metacognition which could and have been applied to multiple forms of interventions including cognitive remediation (Cella et al., 2015); metacognitive training (Moritz et al., 2018), psychoanalysis (Ridenour et al., 2019; Pec et al., 2020), shared decision making (Zisman-Ilani et al., 2021) and social practice (Rice et al., 2020). These basic conditions have been proposed to include joint reflection between the clinician or peer and the identified patient, the rejection of stigmatizing beliefs about mental illness, and flexibility for the identified patients to develop and change their goals as they progress. These principles do not preclude the possibility of a manualized curriculum, but it reminds the field that reflection cannot be scripted or taught. To promote the growth of metacognitive capacity, attention is needed to elicit and develop a persons' unique sense of their own experience of themselves and others, including what transpires in the therapeutic relationship (Lysaker et al., 2020c). These sets of reflections are likely to take any number of turns and are likely to involve both pain and the need for persons to decide what risks they are willing to take to attain wellness (Zisman-Ilani et al., 2021).

This is also not to say social cognitive interventions cannot address metacognition. Indeed, Hasson-Ohayon (2012) has described how one of the prominent approaches to social cognition, SCIT, can be easily infused with the processes that promote metacognition. As in the case of other interventions, as noted above, it remains essential to see that these treatments will not affect metacognitive processes if they remain focused on teaching or “getting” people to do certain things. Reflection and the growth of metacognitive capacity cannot, by definition, be directed by others. If patients are to develop metacognitive capacity and thereby develop a richer sense of the purposes, possibilities and positions of others and then pursue relationships, they must have the space to do so with the genuine input but not control of the identified treater.

The potential for the growth of metacognition and the amelioration of social deficits is finally revealed in this research to be a complex process which calls for more than the development of one's thoughts about others and the community. Theoretically, it is unlikely that it would be sufficient to address only thoughts about other people. It would seem necessary for treatment that might affect metacognition and ultimately social function to consider patients' sense of self and his or her own challenges and possibilities which clearly influence and are influenced by thoughts about others and one's community. Indeed, as suggested in earlier clinical studies, the growth of self-reflectivity may be a condition for the later growth of other metacognitive capacities, including awareness of the other (Lysaker et al., 2007a; Dimaggio et al., 2008).

## Conclusions and Limitations

In summary, models have suggested that social dysfunction among persons diagnosed with psychosis reflects social cognition deficits which lead to systemic failures in understanding others' thoughts, feelings, and intentions. While this view is supported by empirical work and intuitively appealing, it does not fully explain deeper aspects of social dysfunction, including the lack of meaningful connection to others and their community among persons diagnosed with psychosis. In response, this paper has reviewed research on metacognition and suggested that the metacognitive deficits observed in psychosis may result in more than a loss of the ability to correctly understand others emotional and cognitive states. Metacognitive deficits may lead to a failure to develop a dynamic and contextually responsive sense of others' purposes, possibilities and positions in the world. Such a failure would erode the potential for cooperative, mutually understood relationships which go beyond current practical circumstances, thereby creating barriers to healthy intersubjectivity. Metacognitive deficits thus explain more than a set of specific errors. They account for the lack of a larger, contextualized, and holistic understanding of the other as well as the potential for connection over time.

This view has specific implications for psychosocial treatments. In particular, while it has spawned certain treatments (e.g., MERIT; Lysaker and Klion, 2017), it also points to a number of important qualities which are necessary for approaches that might enhance metacognition and therein social function. These include the need for joint meaning making rather than primarily teaching or skill building as directed by a more powerful clinician. This kind of treatment also requires the consideration of multiple facets of metacognition and the natural unpredictability of any kind of truly personally tailored treatment.

Of note, there are limitations and need for continuing research. We have focused on the contributions of one model of metacognitive and its study in psychosis. There are other aspects of metacognition that have been explored in other experimental contexts (Rouy et al., 2021). It would be important to consider these in psychosis in regard to social function in general. The connection of these ideas with other models of the experience of psychosis is also needed (Hurlburt, 1990; Škodlar and Henriksen, 2019).

Moreover, while we have focused on the correct apprehension of other's mental states, other work has grown considering cognitive biases (Buck et al., 2020). Thus, work is needed to continue to examine how these bodies of work interface. Further research also should explore potentially different contributions made by different domains of metacognition in different spheres of social function. Further, existing research is generally cross sectional. Longitudinal work is needed to explore how social cognition, metacognition and social function interact over time. Finally, there are other variables to consider in these complex relationships, including others forms of cognition, trauma, stigma and phenomena deeply related to intersubjectivity such as attachment style and emotional regulation.

## Author Contributions

PL, IH-O, CW, and JL: conceptualization of the paper. PL, IH-O, CW, KH, and AM: literature search. PL, IH-O, CW, KH, AM, and JL: interpretation of the review and writing of the first draft. PL, IH-O, CW, AM, and JL: writing of the final draft. All authors contributed to the article and approved the submitted version.

## Funding

Emory University's College of Arts and Sciences provided the funding to meet the open access publication fees.

## Conflict of Interest

The authors declare that the research was conducted in the absence of any commercial or financial relationships that could be construed as a potential conflict of interest.

## Publisher's Note

All claims expressed in this article are solely those of the authors and do not necessarily represent those of their affiliated organizations, or those of the publisher, the editors and the reviewers. Any product that may be evaluated in this article, or claim that may be made by its manufacturer, is not guaranteed or endorsed by the publisher.
